# Daily Intake of Probiotics with High IFN-*γ*/IL-10 Ratio Increases the Cytotoxicity of Human Natural Killer Cells: A Personalized Probiotic Approach

**DOI:** 10.1155/2014/721505

**Published:** 2014-12-11

**Authors:** Yu-Hsuan Ho, Yu-Chiu Lu, Hung-Cheng Chang, Shin-Yi Lee, Min-Fen Tsai, Yu-Ting Huang, Ting-Yuan Hsu

**Affiliations:** Department of Research and Development, Bio Ray Biotech Inc., 3F., No. 466, Bo'ai 1st Road, Gushan District, Kaohsiung City 80466, Taiwan

## Abstract

A personalized probiotic microfluidic chip system has been established and used to screen the probiotics which had the highest value of IFN-*γ*/IL-10 or IL-10/IFN-*γ* among six probiotics, including *L. paracasei* BRAP01, *L. acidophilus* AD300, *B. longum* BA100, *E. faecium* BR0085, *L. rhamnosus* AD500, and *L. reuteri* BR101. One hundred volunteers were included and their PBMCs were collected and stimulated by the six probiotics. People who belonged to the IFN-*γ* group took the probiotics that exerted the highest ratio of IFN-*γ*/IL-10 and vice versa in IL-10 group. A significant increase in NK cytotoxicity of 69 volunteers in the IFN-*γ* group was observed compared to the IL-10 group (*n* = 21) and control group (*n* = 10). The result also showed that *L. paracasei* BRAP01 and *L. acidophilus* AD300 were the two dominant inducers in IFN-*γ* group which yielded higher value of IFN-*γ*/IL-10 than the other 4 probiotics, while *L. reuteri* BR101 was the most effective agent on the ratio of IL-10/IFN-*γ* in the IL-10 group. Our finding highlighted the concept of personalized probiotics and also provided a good foundation to investigate the probiotics with NK activity.

## 1. Introduction

Probiotics are now widely considered as beneficial bacteria which have various good effects on the human beings; among them,* Lactobacillus* and* Bifidobacterium* are the most common ones [1–4]. The advantages posed by these probiotics carry the potential to be used in clinical practices, such as diabetes, oral health, atopic diseases, liver diseases, urogenital infections, normal immune function, virus infections, and even the anticancer capabilities [1]. Until now, several studies have been conducted to elucidate the relations between colorectal cancer and probiotics. Probiotics are able to maintain the balance of microflora and also decrease the oxidative stress in the gut. Some studies even indicated that the consumption of probiotics is a potential way to prevent the cancer [5, 6].

Peripheral blood mononuclear cells (PBMCs) are mainly composed of T-cells, B-cells, monocytes, and natural killer cells (NKs). These cells play critical roles in maintaining the proper immune function in response to the environmental stimulus. Additionally, PBMCs are also good and easily accessible resources, which makes them become an attracting target for investigating immune system [7]. Among PBMCs, NKs are notable for their tumor killing ability via nonspecific lysis of cancer cells. The phenotype of NKs is defined as CD3^−^, CD16^+^, and CD56^+^. Basically, NK cells protect the host by eliminating the invasion of microorganisms and also inhibiting the process of malignant transformation; thus, the cytotoxicity of NKs should be kept in a normal range [8, 9]. The lower NK cytotoxicity has been connected to the severity of several diseases, such as chronic fatigue immune dysfunction syndrome [10], atherosclerosis [11], myeloproliferative disorders [12], and obsessive-compulsive disorder [13]. Besides, several studies have shown that decreased NK numbers and killing ability are correlated with cancer progress. Sha et al. [14] showed that patients with Hepatitis B cirrhosis and liver cancer had significant lower NK cytotoxicity and number compared with healthy people. Liu et al. [15] have indicated that the metastasis of prostate cancer is positively associated with the loss of peripheral NK cells. Also, higher cytotoxicity of NK cells has been shown to prevent the colorectal cancer risk [16]. Taken together, maintenance of regular NK function is a crucial issue for host.

Until now, several reports have demonstrated that certain strains of probiotics could enhance the NK activities in human trials. In an earlier report, the authors demonstrated that elderly people who consume fermented milk which contained* L. casei* Shirota exhibit elevated NK activity in 3 weeks; however, this phenomenon disappeared after 6 weeks [17]. More recently, Reale et al. [18] showed that the smokers can restore their NK function if they have daily intake of* L. casei* Shirota within 3 weeks [18]. These data suggested that probiotics may have positive effects on the NK functions; however, these studies only included one probiotic strain and it seems that the efficacy of the probiotics decreased when the time is prolonged (3 weeks). Consequently, the current knowledge about the correlation between NK and probiotics is still incomplete and more studies are needed to be conducted to confirm this phenomenon.

In the present study, we attempted to observe the efficacy of taking personalized probiotics on NK activity based on the ratio of IFN-*γ* to IL-10.

## 2. Materials and Methods

### 2.1. Participants

The blood samples were collected from 100 volunteers. For comparison, people were divided into 3 groups which were IFN-*γ*, IL-10, and control group. The numbers and average age of participants in these three groups are as follows: IFN-*γ*: *n* = 69, age ≈ 55; IL-10: *n* = 21, age ≈ 57; control: *n* = 10, age ≈ 53. In IFN-*γ* group, the PBMCs from participants were stimulated with 6 probiotic strains and they were asked to take the specific probiotic capsule; this probiotic showed the highest ability to stimulate the secretion of IFN-*γ* based on the ratio of IFN-*γ* to IL-10. In contrast, people in IL-10 group took the probiotics that exhibited the highest IL-10 to IFN-*γ* ratio, while the control group had no consumption of probiotics. The volunteers in both IFN-*γ* and IL-10 groups were asked to take 2 capsules of probiotics one day. Every capsule contains 10^10^ cfu/capsule of probiotics. This study was performed in accordance with the Declaration of Helsinki and approved by the Ethical Committee of Bio Ray Biotech Inc.

### 2.2. PBMCs Stimulation by Six Probiotics

Six probiotic strains were chosen as stimulators on PBMCs. The six strains are* L. paracasei* BRAP01 (Bio Ray Biotech),* L. acidophilus* AD300 (Biena),* B. longum* BA100 (Biena),* E. faecium* BR0085 (Synbio),* L. rhamnosus* AD500 (Biena), and* L. reuteri* BR101 (Bio Ray Biotech). Ficoll-Paque (GE) separation method was conducted to isolate the PBMCs from the blood. Briefly, 10 mL of blood was diluted to 14 mL with PBS and then the diluted blood was carefully layered on top of the Ficoll-Paque solution. The tubes were centrifuged at 500 g for 20 minutes in RT. After centrifugation, the PBMCs were separated from the blood cells and stored in PBS after one-time wash by PBS. The purified PBMCs were cultured with 6 different probiotics in RPMI-1640 (GE) medium with 10% FBS (Gibco), 1X Glutamine (Biowest), and PSN (Gibco) in a 96-well culture plate (SPL). The plate was put in a CO_2_ incubator for 40 hours (37°C, 5% CO_2_). Finally, the plate was centrifuged at 250 g and the supernatant was collected and stored in −20°C.

### 2.3. IFN-*γ* and IL-10 Detection by Microfluidic Chip

To avoid the uncertainty of experiments, we used an automatic and customized microfluidic system (Agnitio BioIC System) to detect the amount of IFN-*γ* and IL-10 secreted by PBMCs. For detection, 200 *μ*g/mL anti-IFN-*γ* antibody (BD) and 400 *μ*g/mL anti-IL-10 antibody (BD) were immobilized in the chips and the enzyme-linked immunosorbent assay (ELISA) was performed to detect IFN-*γ* and IL-10. In brief, all the working solutions were prepared before going through the automatic process. For each solution, there existed a corresponding well to add the buffers of the chip. Conjugate buffer contains 1000-fold dilutions of 0.5 mg/mL biotinylated anti-IFN-*γ* antibody, 0.5 mg/mL biotinylated anti-IL-10 antibody, and 1000X streptavidin-HRP (BD). Sample buffer was the supernatant of PBMC which was cocultured with different probiotic strains. Substrate buffer was SuperSignal ELISA Femto Maximum Sensitivity Substrate (Thermo). Washing and blocking buffers were provided by the manufacturer (Agnitio Science Technology). The volume of each solution was according to the instructions from the manual. When buffers were loaded to each well, the chip was inserted into the Agnitio BioIC Pumping Machine. The machine was prewarmed to 37°C and then automatically started the ELISA process. When washing step was finished, the chip was analyzed by the Agnitio BioIC Analyzer.

### 2.4. NK Cell Mediated Cytotoxicity Measurement

To assess the cytotoxicity of NKs from PBMC, we measured the lactate dehydrogenase (LDH) released from target cells to determine the cytotoxicity of effector cell to target cell [19] and the kit was purchased from Promega. In this experiment, the cancer cell line K562 was target cell to evaluate the cytotoxicity of NK cell (effector cell).

The experiment includes several control groups to be the reference of this assay and the incubation buffer is RPMI-1640 (GE) with 5% FBS (Gibco). Each group is as described below:

1. the effector spontaneous control group: PBMCs only,
2. the target spontaneous control group: K562 cell only,
3. the total lysis group: K562 cell and lysis buffer,
4. the volume correct group: incubation buffer with lysis buffer,
5. experimental group: PBMCs and K562 cell.

In the beginning, the PBMCs were cocultured with K562 at a ratio of 25 : 1 (effector cell/target cell) at 37°C with 5% CO_2_ for 4 hours in a 96-well plate. After incubation, the plate was centrifuged at 250 g for minutes and then 50 *μ*L aliquot of supernatant was transferred to a 96-well ELISA plate (SPL). The aliquot was mixed with LDH substrate mix solution for 20 minutes. Finally, add 50 *μ*L of stop solution to each well and measure the OD_492_ with an ELISA reader (TECAN Infinite M200). The calculation of cytotoxicity percent of PBMCs to K562 is performed using the following formula: cytotoxicity percent % = (experimental group − effector spontaneous group − target spontaneous group)/(total lysis group − volume correct group − target spontaneous group) ∗ 100%.

### 2.5. Statistical Calculation and Data Analysis

The data analysis was performed in Perl 5.0 and the statistical calculation was done in R 3.0. Significant calculation for comparing the NK cytotoxicity was calculated via Wilcoxon signed rank test.

## 3. Results

### 3.1. Microfluidic Chip Assays

**Figure 1:**
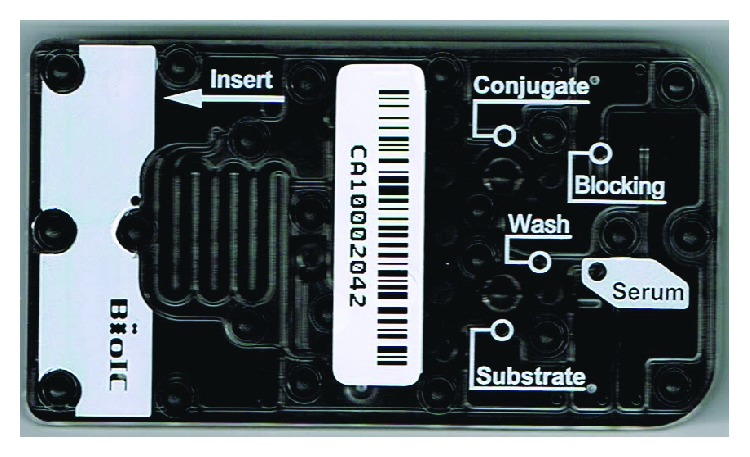
A figure of the microfluidic chip. After adding buffers to each well, the chip then went through the automatic process in a reaction chamber. The entire process was about 1 hour and then the chip can be taken out to the analyzer for determining the amount of IFN-*γ* and IL-10.

The first process of this study is to determine the personalized probiotics from six probiotic strains (*L. paracasei* BRAP01,* L. acidophilus* AD300,* B. longum* BA100,* E. faecium* BR0085,* L. rhamnosus* AD500, and* L. reuteri* BR101) for each participant by the microfluidic chip assay. The microfluidic chip was displayed in Figure 1; compared to the traditional ELISA process, this platform avoids the manual error because the reactions of each process were automatically performed. Thus, manual errors or influences can be limited. Typical microfluidic results are displayed in Table 1. This table displays the percentage of IFN-*γ*/IL-10 or IL-10/IFN-*γ* ratio of six probiotics for specific subjects. For instance, in the left part of Table 1, the subject took the* L. paracasei* BRAP01 as his own personalized probiotics, while, in the right part, the person took the* L. reuteri* BR101 for comparison. By doing so, people in IFN-*γ* group consumed the probiotics that had the highest effect on the production of IFN-*γ* and this condition was opposite in the IL-10 group. The purpose of this experimental design is to separate the subjects into two different groups and we want to clarify the influence on NK activity based on these two totally different approaches.

### 3.2. NK Cell Activity after Six Months

The observation of the efficacy of taking personalized probiotics on the average NK cytotoxicity of all the participants was displayed in Table 2. It is clear that, after six months, a significant increase was noticed in IFN-*γ* group. The cytotoxicity value raised around 6.3% and the *P* value was lower than 0.005. In IL-10 group, a slightly increasing trend can be seen (~1.4%); however, the *P* value (0.117) showed no statistical difference between the first test and the second test. For control, the value dropped about 3% in six months.

In the IFN-*γ* group, some of them even showed a substantial increase compared to the first time (over 20%). In control group, the change seems not obvious within six months, since the SD of variation of NK cytotoxicity was much lower than the IFN-*γ* group. In the IL-10 group, a mild increase in the NK cytotoxicity was observed; however, in comparison to IFN-*γ* group, the raising performance was much lower than that. From this analysis, it is distinctly indicated that people's taking of the probiotics with the highest ratio of IFN-*γ*/IL-10 leads to a significant increase in their NK cytotoxicity against K562 cancer cell line. In the previous reports, researchers have demonstrated that, after three weeks, the effectiveness of probiotics on NK activity was vanished [17, 18]; however, our results showed that the efficacy of probiotics was able to persist in six months. This indicated that the personalized probiotics based on the ratio of IFN-*γ*/IL-10 strategy could be a promising methodology to activate or restore the tumor killing capability of human peripheral immune system.

### 3.3. Personalized Probiotics Profile

In addition to the global analysis of the NK activity before and after the probiotics consumption, the contributions of individual probiotics were also analyzed. The summary of NK cytotoxicity change with probiotics is displayed in Table 3; for IFN-*γ* group,* L. paracasei* BRAP01 and* L. acidophilus* AD300 account for a large portion of the entire group and the percentage of* L. reuteri* BR101 was zero. In contrast,* L. reuteri* BR101 constituted about 48% in IL-10 group, while the* L. paracasei* BRAP01 and* L. acidophilus* AD300 were not observed in the IL-10 group. For* E. faecium* BR0085, it had only 3% in IFN-*γ* group and increased to 29% in the IL-10 group.* L. rhamnosus* AD500 was relatively even distributed in both groups compared to other strains. From our bacteria profiles, it is apparent that* L. paracasei* BRAP01 and* L. acidophilus* AD300 tend to induce the IFN-*γ* production from PBMCs. Oppositely,* L. reuteri* BR101 and* E. faecium* BR0085 are the potent agents to make PBMCs produce IL-10.

From previous studies,* L. paracasei* and* L. acidophilus* have been shown to improve the allergy status of humans such as rhinitis [20, 21]. Our result also suggested that* L. paracasei* and* L. acidophilus* are two dominant probiotics to induce the IFN-*γ* production, and IFN-*γ* has been reported to inhibit the Th2 cytokine production which is considered to cause the allergic inflammation [22]. Our results were in agreement with these studies, since we also showed that these 2 strains were effective IFN-*γ* inducer.

In a mouse model study, they demonstrated that* L. reuteri* compensated the anti-inflammation function of IL-10 lacking mouse suggesting that this bacteria can restore Il-10 competency for host [23]. Yet another study indicated that the* L. reuteri* RC-14 exerted downregulation of IL-10 in the patients with neurogenic bladder of spinal cord injury [24]. These results showed that* L. reuteri* may exhibit different immune regulatory effects under diverse conditions, while, in this study, we showed that* L. reuteri* was the most efficient one to induce IL-10 other than IFN-*γ* among the six strains.

Except for the dominant probiotics for IFN-*γ* or IL-10 groups, the other probiotics,* B. longum*,* E. faecium*, and* L. rhamnosus*, were presented in both IFN-*γ* and IL-10 group suggesting the bidirectional roles for immune regulation that they may play, which means the same probiotic strain may have particularly dissimilar influence on different persons.

### 3.4. Different Probiotic Effects on the NK Killing Ability

**Figure 2:**
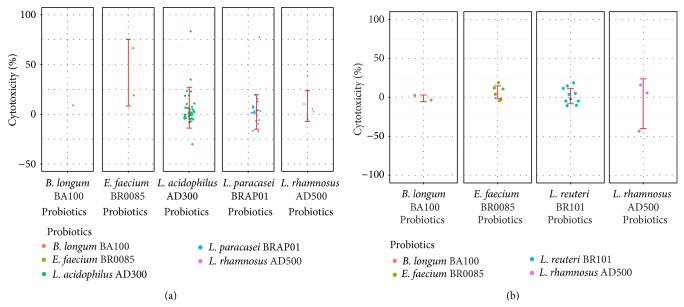
Distribution of the probiotic effects on the NK cytotoxicity variations. (a) IFN-*γ* group. (b) IL-10 group. Some probiotics exhibited good potential to enhance the NK activity, such as* E. faecium* BR0085 in both groups. The probiotics in IFN-*γ* group possessed a significant capability to increase the NK cytotoxicity compared to the IL-10 group.

Besides the analysis of occurrence of each probiotic, Table 3 also displays the overall performance of 6 probiotics on the NK activity. As mentioned in previous section, IFN-*γ* group mediated a significant increase in NK cytotoxicity but not in IL-10 group. It is clear that the average NK cytotoxicity variations of 5 strains in IFN-*γ* group were all positive (except for* L. reuteri* BR101, which was zero occurrences). In the IL-10 group, only* E. faecium* BR0085 showed greater ability to increase the NK cytotoxicity and the other strains were all below the average value (1.4%). Some of them were even negatively correlated with the NK cytotoxicity tendency (*B. longum* BA100: −1.33%,* L. rhamnosus* AD500: −7.66%). Detailed variations of the probiotics effects on NK activity were displayed in Figure 2(a) (IFN-*γ* group) and Figure 2(b) (IL-10 group).

In Figure 2, although a larger portion of probiotics showed great potency to increase NK cytotoxicity, the NK activity seemed not improved of some participants. However, by comparison of IFN-*γ* and IL-10 group, it is still an evident distinctness between these two groups. An apparent increasing tendency was observed in IFN-*γ* group, while this phenomenon disappeared in IL-10 group.

In IFN-*γ* group,* E. faecium* BR0085 exhibited promising ability to increase the NK cytotoxicity and the average value is 41.8%. This value is about 7 times larger than the average value of all 6 probiotics (6.3%); however, the sample size is too small for* E. faecium* BR0085 (only 2). Thus, it could be argued that this phenomenon may disappear when the subject number increases. In IL-10 group, the overall increasing percent of NK cell cytotoxicity of* E. faecium* BR0085 is 6.74%. Again,* E. faecium* BR0085 was still the most effective agent (*n* = 6) to stimulate the NK activity in IL-10 group as shown in Figure 2(b). Consequently, the probiotic strain,* E. faecium* BR0085, showed a good potential to stimulate the cancer killing ability of NK cells and this mechanism may not rely on the production of IFN-*γ* or IL-10; however, more evidence is needed to support this observation.

## 4. Discussion and Conclusions

In this pilot study, we showed that taking the probiotics with the highest value of IFN-*γ*/IL-10 can enhance the cytotoxicity of NK cells after six months. This result also suggested that even the same strains of probiotics possibly have different or opposite effects on the hosts. Thus, it should be noted that a deep evaluation of probiotics is required when we want the certain effect of a probiotic, since it could be useful to some people but useless to others.

As mentioned before, some studies have been conducted to show the advantages of probiotics in elevating the NK function in mouse and human models [25, 26]; however, there is no comprehensive study to investigate the relations between probiotics and NK cell activity. Herein, we showed that people who took the probiotics which had the highest value of IFN-*γ*/IL-10 among 6 strains will also have significantly higher NK activity after six months in comparison to the other 2 groups (IL-10 and control group). Based on the results, we proposed that the probiotics which are highly active to induce the IFN-*γ* production other than IL-10 from host have great potency to become effective NK activated agents. Several papers have reported that the NK activity was highly related to the amount of IFN-*γ*. A survival study on gastrointestinal stromal tumor-bearing patients revealed that the higher NK cell and IFN-*γ* production was a predictor for a long term survival of these patients [27]. Another paper showed that the decreased NK cytotoxicity and IFN-*γ* level were associated in breast cancer patients and this decrement also related to the progression of this disease [28]. IFN-*γ* has been characterized to enhance the NK cell cytotoxicity by increasing the tumor cell's sensitivity to the NK mediated killing process [29]. In fact, NK cell can release IFN-*γ* to stimulate the dendritic cells (DCs) to produce IL-12, and then IL-12 will amplify the IFN-*γ* production from NK cell. This feedback process constitutes the innate immune response [30]. Besides IFN-*γ*, a paper demonstrated that, under the systemic infections by pathogens, NK cell released IL-10 to inhibit the IL-12 production by DCs [31]. Another study demonstrated that, under the bacteria infection, IL-10 suppressed the NK activity [32]. Taken together, we proposed that the rationale for probiotics to trigger NK cell mediated cytotoxicity is via the indirect effect. For example, when DC cells are under the stimulation of highly active IFN-*γ* producing probiotics, this may lead to the secretion of IL-12 by DCs which can maintain a proper feedback loop of DC to NK.

In conclusion, this paper attempted to obtain a comprehensive understanding of the effects of probiotics on NK activity. Although we successfully showed that people who took the probiotics with higher IFN-*γ*/IL-10 value could enhance the NK cytotoxicity, some people in the IFN-*γ* group had decreasing NK activity suggesting that the variation of immune response among people is remarkable. This may be due to the insufficient dosage of probiotics taken by participants or because the probiotics we chose were not suitable for them. However, this pilot study demonstrated that the consumption of probiotics for individuals should be carefully concerned and the value of IFN-*γ*/IL-10 could be a potential biomarker to evaluate the usage of probiotics to some extent.

## Figures and Tables

**Table 1:** Representative results of microfluidic assays in IFN-*γ* and IL-10 groups.

IFN-*γ* group	IFN-*γ*/IL-10 (%)	IL-10 group	IL-10/IFN-*γ* (%)
***L. paracasei *BRAP01**	**56.2 **	*L. paracasei *BRAP01	2.7
*L. acidophilus *AD300	24.8	*L. acidophilus *AD300	0.5
*B. longum *BA100	5.2	*B. longum *BA100	8.7
*E. faecium *BR0085	3.9	*E. faecium *BR0085	34.2
*L. rhamnosus *AD500	7.5	*L. rhamnosus *AD500	3.2
*L. reuteri *BR101	2.4	***L. reuteri *BR101**	**50.6 **

**Table 2:** The comparison of three groups between the first NK activity assessment and after six months. The asterisk symbol denotes the significant difference by Wilcoxon signed rank test.

Group category	First value	Value after six months	Increasing percentage	SD of first value	SD after six months	*P* value of six months to first month	Percentage of increasing NK cytotoxicity >20%

IFN-*γ*	11.76%	18.06%	6.30%	10.08%	17.5%	0.003^*^	10.15%
IL-10	14.12%	15.52%	1.40%	14.12%	13.26%	0.117	0%
Control	14.90%	11.83%	−3.07%	13.97%	7.51%	NA	0%

**Table 3:** Profiles of probiotics after microfluidic assays and the corresponding NK activity change for each probiotic after six months.

Group category	Number	Occurrence percentage	Average increasing NK cytotoxicity	SD of NK cytotoxicity
**IFN-** **γ**	69	100%	6.3%	18.2%
*B. longum *BA100	1	1%	8.09%	None
*E. faecium *BR0085	2	3%	41.80%	None
*L. reuteri *BR101	0	0%	None	None
*L. rhamnosus *AD500	5	7%	9.60%	16.80%
*L. acidophilus *AD300	39	57%	4.34%	16.57%
*L. paracasei *BRAP01	22	32%	4.86%	18.30%

Group category	Number	Occurrence percentage	Average increasing NK cytotoxicity	SD of NK cytotoxicity

**IL-10**	21	100%	1.4%	13.6%
*B. longum *BA100	2	9%	−1.33%	None
*E. faecium *BR0085	6	29%	6.74%	7.90%
*L. reuteri *BR101	10	48%	1.58%	9.90%
*L. rhamnosus *AD500	3	14%	−7.66%	31.90%
*L. acidophilus *AD300	0	0%	0.00%	None
*L. paracasei *BRAP01	0	0%	0.00%	None
